# The Effect of Brief Stair-Climbing on Divergent and Convergent Thinking

**DOI:** 10.3389/fnbeh.2021.834097

**Published:** 2022-01-28

**Authors:** Karin Matsumoto, Chong Chen, Kosuke Hagiwara, Natsumi Shimizu, Masako Hirotsu, Yusuke Oda, Huijie Lei, Akiyo Takao, Yuko Fujii, Fumihiro Higuchi, Shin Nakagawa

**Affiliations:** Division of Neuropsychiatry, Department of Neuroscience, Yamaguchi University Graduate School of Medicine, Ube, Japan

**Keywords:** acute aerobic exercise, stair-climbing, creativity, divergent thinking, alternate use test, insight problem-solving

## Abstract

Recent studies show that even a brief bout of aerobic exercise may enhance creative thinking. However, few studies have investigated the effect of exercise conducted in natural settings. Here, in a crossover randomized controlled trial, we investigated the effect of a common daily activity, stair-climbing, on creative thinking. As experimental intervention, subjects were asked to walk downstairs from the fourth to the first floor and back at their usual pace. As control intervention, they walked the same path but using the elevator instead. Compared to using the elevator, stair-climbing enhanced subsequent divergent but not convergent thinking in that it increased originality on the Alternate Use Test (*d* = 0.486). Subjects on average generated 61% more original uses after stair-climbing. This is the first study to investigate the effect of stair-climbing on creative thinking. Our findings suggest that stair-climbing may be a useful strategy for enhancing divergent thinking in everyday life.

## Introduction

Creativity or creative thinking, a high-level cognitive function, is the key to invention and innovation in many fields of human society, including but not limited to science, technology, industry, business, education, and art (Hennessey and Amabile, 2010; Sternberg and Kaufman, 2018). The development of effective tools to evaluate and strategies to enhance creative thinking, therefore, has attracted much attention in the past decades (Guilford, 1967b; Hennessey and Amabile, 2010; Sternberg and Kaufman, 2018). Creative thinking comprises two fundamental processes: divergent and convergent thinking. Whereas the former involves stretching beyond existing solutions to generate multiple, novel ones, the latter involves approaching a single correct, objective-appropriate solution (Guilford, 1967a). It has been shown that performance on tests of divergent and convergent thinking predicts creative potential and achievement in real-life as well as creativity evaluated by others (Cropley, 2000; Kim, 2008; Runco et al., 2010; Gralewski and Karwowski, 2019).

A recent, timely systematic review concluded that a single bout of aerobic exercise may enhance creative thinking (in particular divergent thinking, Aga et al., 2021). In the reviewed studies, divergent thinking was typically evaluated with the original or adapted versions of the Alternate Uses Test (AUT, Guilford, 1960; Torrance, 1966). In this test, subjects have to write down as many as possible unusual uses of common objects, such as “bricks.” The number of generated uses (known as fluency), the number of conceptual categories the generated uses are from (flexibility), and the rareness of the uses (originality) are commonly employed as indicators of divergent thinking (Runco and Acar, 2012; Reiter-Palmon et al., 2019). Studies conducted in the laboratory have reported that aerobic workout or dance for approximately 20 min (Steinberg et al., 1997), walking on a treadmill for 4 min at a self-selected pace (Oppezzo and Schwartz, 2014) or 44 min at vigorous-intensity (Netz et al., 2007), or cycling on an ergometer for 15 min at roughly light to moderate intensity (Aga et al., 2021) enhanced one or multiple indicators of divergent thinking. Convergent thinking, in contrast, was unaffected or uninvestigated in these studies.

Despite these encouraging findings, whether aerobic exercise commonly conducted in everyday life enhances creative thinking remains unclear. To our knowledge, only two studies have investigated the effect of acute aerobic exercise conducted in natural, real-life settings. One study investigated the effect of walking through a university campus (Oppezzo and Schwartz, 2014), and the other the effect of a 45-min physical education class featuring aerobic games (Román et al., 2018), both of which reported enhanced divergent thinking after the intervention. Neither studies, however, evaluated convergent thinking.

In the present study, therefore, we aimed to advance our understanding of the impact of aerobic exercise conducted in everyday life on both divergent and convergent thinking, by focusing on a common physical activity, stair-climbing. Climbing stairs at comfortable or fast paces and with or without carrying groceries or other loads, has been a recommended physical activity in many governmental guidelines, such as those of the United States (US Department of Health and Human Services, 2018) and Japan (Japanese Ministry of Health Labour and Welfare [JMHLW], 2013). Based on previous reports that stepping over obstacles and precision stepping activate the prefrontal cortex (PFC), a primary neural substrate of divergent thinking, cognitive flexibility, and executive functions in general (Yuan and Raz, 2014; Dajani and Uddin, 2015; Wu et al., 2015), we hypothesized that a brief stair-climbing intervention enhances divergent and convergent thinking.

## Materials and Methods

### Participants

The study was approved by the authors’ Institutional Review Board and carried out in accordance with the Declaration of Helsinki. All subjects provided written informed consent. The study was also preregistered on the University hospital Medical Information Network Clinical Trial Registry (UMIN-CTR). Using data from Oppezzo and Schwartz (Oppezzo and Schwartz, 2014) that reported an effect size of *d* = 0.70 (Experiment 1, Walking versus Siting within-subjects), we estimated that to detect such an effect size with power = 0.8, alpha = 0.05, two-sided, 19 subjects were necessary. Considering dropout cases, we recruited 22 subjects (12 males, 10 females, including 19 medical undergraduates and three graduate students; age: 21.36 ± 1.33 years). The inclusion criterion was being in their twenties. The exclusion criteria were reporting any current psychiatric disorders (or currently psychiatric examinations) and having participated our previous studies of creative thinking.

### Design and Procedure

Before the laboratory visit, subjects received instructions to get enough sleep and refrain from doing intense physical activities and from smoking and consuming drinks with caffeine for at least 2 h before coming to the laboratory. They were also asked to change the experimental schedule if they were sick.

Upon arriving, subjects first provided written informed consent after receiving a detailed description of the study. They then filled out questionnaires and answered their demographic information together with a few questions to check if they have followed the above instructions. Subjects also indicated their baseline mood using the Positive and Negative Affect Schedule [PANAS, Watson et al., 1988].

**FIGURE 1:**
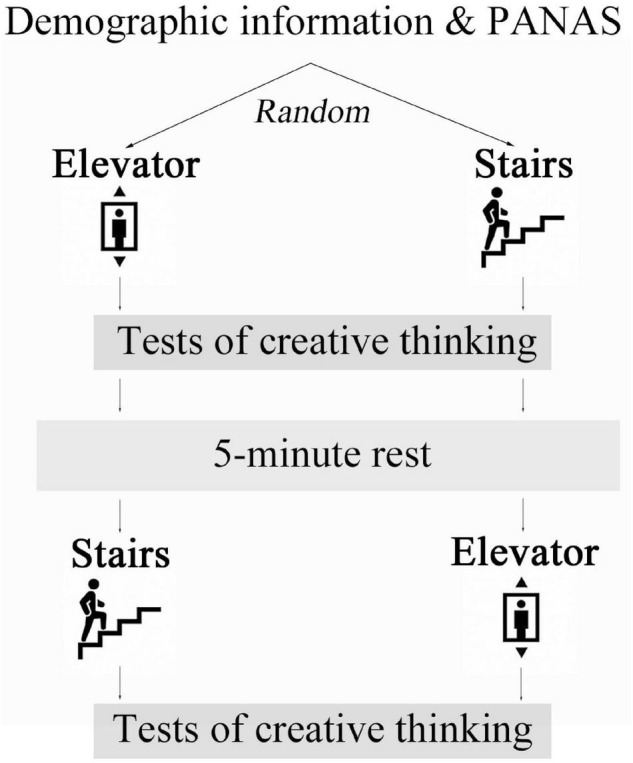
Schematic illustration of the procedure.

The study was a crossover randomized controlled trial with a posttest comparison design (Figure 1). Subjects were assigned to receive a brief stair-climbing and control intervention in a counter balanced order and immediately after each intervention they conducted tests of creative thinking. The trial was repeated on two separate days to investigate the effect on divergent and convergent thinking, respectively. The order of the divergent and convergent thinking tests was also randomized. Statistical comparisons showed that subjects reported similar baseline positive affect (paired *t*-test, *t* = −0.697, *p* = 0.493) and negative affect (Wilcoxon signed-rank test, *Z* = −1.267, *p* = 0.205) on the two test days, suggesting that the randomization was appropriate.

During both interventions, subjects wore an Apple Watch (Series 4, Apple Inc.) to measure their heart rate and time taken to finish the intervention. The accuracy of Apple Watch for the measurement of heart rate has been established (Wang et al., 2017). Immediately after each intervention, subjects also indicated their current mood on a visual analog scale in terms of pleasure, relaxation, and vigor (Aga et al., 2021). As a washout period, after the first phase of intervention and creative thinking test, subjects rested for 5 min. After finishing the last creative test on the second test day (i.e., at the end of the experiment), subjects also filled out the International Physical Activity Questionnaire (IPAQ) to report their levels of vigorous physical activity, moderate physical activity, and walking in the past 7 days.

### Intervention

For the stair-climbing intervention, starting from the laboratory room on the fourth floor, subjects were asked to walk downstairs to the first floor and back after approaching but without stepping out of the entrance of the building. A schematic illustration of this round-trip path and the layout of the first and fourth floors is shown in Supplementary Figure 1. One flight had 21 stairs in between. For the control intervention, they were asked to walk the same path but using the elevator instead. Subjects were asked to walk at their usual pace for both interventions. They were also asked to remain quiet and not to speak with anyone they might encounter throughout the interventions.

The rationale of selecting this intensity of exercise intervention (i.e., walking downstairs of three flights and then back) was three-fold. Firstly, this intensity (in Metabolic equivalents) was roughly equal to that of treadmill walking for 4 min as used in Oppezzo and Schwartz (Oppezzo and Schwartz, 2014), as estimated based on our pilot testing. Secondly, we could also have set the walking distance to five flights since we also had laboratory rooms on the sixth floor; however, in our pilot testing, subjects felt somewhat tired and short of breath, which did not allow us to start the creative thinking tests immediately after the intervention. Thirdly, based on our pilot survey with staffs and students in our department, most people tended to use stairs for two or three flights.

The building in which we conducted the experiment was a research building with six floors and nine clinical departments. The elevator was most crowded before 9:00 in the morning and after 17:00 in the afternoon because of the commuting traffic, and was moderately crowded between 12:00 and 14:00 because of the lunch break. The experiment was conducted between 9:00–12:00 am and 14:00–17:00 pm, during which time the traffic was generally considered mild.

### Divergent Thinking

The commonly employed AUT was used to evaluate divergent thinking. In this test, given 4 min, subjects had to think of original and unusual uses of three common objects (e.g., “brick”) and write their answers on a blank paper sheet of A4-size. Different objects were used for each phase of intervention, the order of which was also randomized for each subject.

Following previous studies, fluency, flexibility, and originality were employed as indicators of divergent thinking (Runco and Acar, 2012; Reiter-Palmon et al., 2019). Fluency was defined as the total number of unusual uses (excluding the common use of each object). Flexibility was defined as the total number of conceptual categories the uses are from (e.g., “dumbbell” and “objects for muscle training” belong to the same conceptual category). Originality was the rareness of the uses and here defined according to the conceptual category of the uses. That is, only if a single participant generated use(s) from a specific conceptual category, that category was considered original; if two participants generated use(s) from the same conceptual category, the category was considered original for neither of them.

Before scoring all responses, the primary coder (i.e., the first author) first received extensive training with the scoring method. Using data from our previous study, the primary coder reached almost perfect agreement (Cohen, 1960) with our previous coder, as indicated by Cohen’s κ = 0.970 and 0.812 for flexibility and originality, respectively. After training, the primary coder scored all participants while staying blinded to the intervention order. To further ensure reliability, we also asked a secondary coder to score responses for a randomly selected object. The two coders reached an agreement of κ = 1.000 and 0.789 for flexibility and originality, respectively.

### Convergent Thinking

The matchstick arithmetic problems created by Knoblich et al. (1999) were used to measure convergent thinking. Each problem was shown as an incorrect equation written with Roman numerals made by matchsticks and subjects had to move one stick to make the equation correct. For instance, for the problem of IV = III + III, the correct answer was to move one stick from the left side of “IV” to its right side to form “VI.” Three different types of problems depending on the way of moving the matchstick were selected for each set of divergent thinking test (administered after each phase of intervention). Based on our pilot testing, the time limit was set to 4 min. The number of correctly solved problems was used as the indicator of convergent thinking.

At the beginning of the experiment on the test day of convergent thinking, all subjects were first trained to be able to recognize the Roman numerals. After they finished the second phase of convergent thinking test, subjects were also asked if they had ever seen any of the matchstick problems tested. No subjects had seen exactly the same problems before the study.

### Statistical Analysis

The statistical analysis was conducted with IBM SPSS Statistics 26.0. Due to non-normal distribution based on the Shapiro–Wilk test, Wilcoxon signed-rank tests were used to compare divergent and convergent thinking indicators after the stair-climbing versus control intervention. Two-way repeated measures ANOVAs were used investigate the effect of the test day (divergent versus convergent thinking) and the intervention (stair-climbing versus control) on heart rate, time taken to return to the laboratory room, and post-intervention mood. G*Power Version 3.1.9.7 (Faul et al., 2007) was used for calculating effect sizes. *P* < 0.05 was considered significant.

## Results

### Heart Rate, Time Taken, and Mood Measures

**FIGURE 2:**
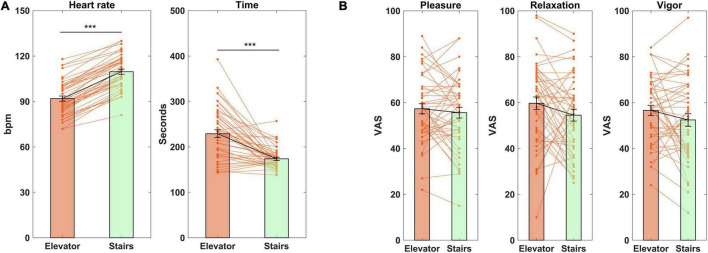
Intervention effect on heart rate, time taken to return to the laboratory room, and mood. **(A)** Heart rate and time taken; **(B)**, mood. The data on different test days are combined and plotted together. ***p < 0.001 bpm, beat per minutes. VAS, visual analog scale. Data shown as mean ± SE.

As reported in Table 1, two-way repeated measures ANOVAs indicated a significant effect of intervention on heart rate (*p* < 0.001) and time taken to return to the laboratory room (*p* < 0.001), while the effect of test day and the intervention and test day interaction remained insignificant. The data on different test days were therefore combined together and plotted in Figure 2A. As can be seen, compared to using the elevator, stair-climbing increased heart rate (109.66 ± 11.198 versus 91.89 ± 11.418 bpm, *d* = 3.547) and cost less time (173.82 ± 23.890 versus 228.98 ± 57.760 s, *d* = 0.937). There were no significant effect of the intervention, test day, or their interaction on the post-intervention mood measures (Figure 2B and Table 1).

**TABLE 1:** The results of two-way repeated measures ANOVAs of heart rate, time taken to return to the laboratory room, and post-intervention mood measures.

	Heart rate	Time taken	Pleasure	Relaxation	Vigor
Intervention	F = 366.328, p = 0.000***	F = 44.112, p = 0.000***	F = 0.516, p = 0.481	F = 2.008, p = 0.171	F = 2.171, p = 0.155
Test day	F = 0.558, p = 0.463	F = 0.824, p = 0.374	F = 0.366, p = 0.551	F = 0.485, p = 0.494	F = 0.002, p = 0.966
Interaction	F = 0.246, p = 0.625	F = 0.883, p = 0.358	F = 3.008, p = 0.098	F = 0.520, p = 0.479	F = 0.145, p = 0.707

***p < 0.001. Statistically significant results are shown in bold.

### Divergent and Convergent Thinking

**FIGURE 3:**
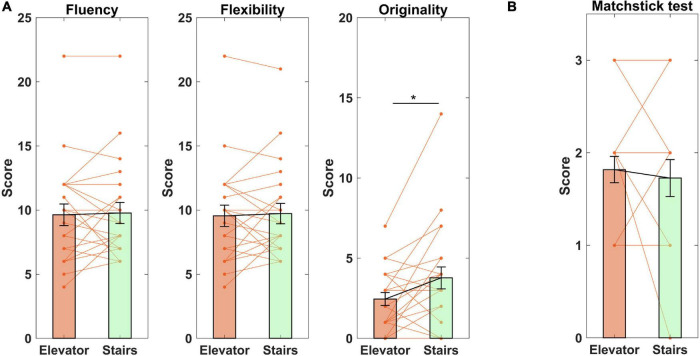
Intervention effect on divergent and convergent thinking. **(A)** Alternate Uses Test; **(B)**, Matchstick test. *p < 0.05. Data shown as mean ± SE.

Scatterplots of the data of divergent and convergent thinking are shown in Figure 3. Wilcoxon signed-rank tests indicated that compared to using the elevator, stair-climbing significantly increased originality (*Z* = 1.977, *p* = 0.048, *d* = 0.486) but not fluency (*Z* = 0.164, *p* = 0.870, *d* = 0.056) or flexibility (*Z* = 0.196, *p* = 0.845, *d* = 0.076) on the AUT. Thus, stair-climbing increased divergent thinking not because subjects thought of more uses (as there was no significant difference in fluency) but because they thought of more original uses. To further verify this conclusion, we divided the number of original uses by the number of total uses generated for each subject. After using the elevator, subjects produced 2.3 ± 1.461 original uses for every 10 generated uses. In contrast, after stair-climbing, they produced 3.7 ± 2.246 original uses for every 10 generated uses. This difference was significant as indicated by a Wilcoxon signed-rank test (*Z* = 2.207, *p* = 0.027, *d* = 0.532). In other words, compared to using the elevator, subjects on average generated 61% more original uses after stair-climbing. With regards to convergent thinking, subjects’ performance on the matchstick test did not differ after using the elevator versus after stair-climbing (*Z* = 0.428, *p* = 0.669, *d* = 0.085). Lastly, we also investigated if the effect of stair-climbing on originality was associated with subjects’ regular physical activity level. The results, however, indicated no significant correlations between the change of originality and total physical activity, vigorous physical activity, moderate physical activity, or walking (all *p* > 0.503).

## Discussion

To our knowledge, this is the first study to investigate the effect of stair-climbing on creative thinking. We found that compared to using the elevator, stair-climbing enhanced subjects’ originality on the AUT, indicating improved divergent thinking. Subjects on average generated 61% more original uses after stair-climbing than after using the elevator. In contrast, stair-climbing did not affect convergent thinking as evaluated by the matchstick arithmetic problems. As a daily physical activity, stair-climbing increased subjects’ heart rate more than using the elevator (*d* = 3.547). Using the commonly employed formula (220-age) as an estimation of maximum heart rate and considering the average age of all subject was 21.36 years, the intensity of the stair-climbing here (with a mean heart rate of 109.66 bpm) was equal to 55% maximum heart rate, or very light (American College of Sports Medicine [ACSM], 2013). Our results suggest that stair-climbing in very light intensity in a natural, real-life setting may enhance divergent thinking. Furthermore, in our experiment, because of the waiting time to use the elevator, stair-climbing also cost less time (*d* = 0.937). Thus, compared to using the elevator, at least for people at the fourth floor, stair-climbing may be a preferred moving method because of its higher efficiency and divergent thinking-enhancing effect.

Among the three measures of divergent thinking, stair-climbing enhanced originality but not fluency or flexibility. Whereas the explanation of such result is unclear, one possibility is that stair-climbing did not change vigor or arousal level in the present study and the latter, however, is required for the enhancing of fluency and flexibility (Aga et al., 2021). Nevertheless, compared to fluency and flexibility, originality is generally considered more important and the key component of divergent thinking (Runco and Acar, 2012; Oppezzo and Schwartz, 2014).

Based on previous reports that stepping over obstacles and precision stepping activate the prefrontal cortex (PFC), a primary neural substrate of divergent thinking, cognitive flexibility, and executive functions in general (Yuan and Raz, 2014; Dajani and Uddin, 2015; Wu et al., 2015), we hypothesized that stair-climbing enhances divergent and convergent thinking. Our hypothesis, however, was only partially supported. Our results are consistent with Oppezzo and Schwartz (Oppezzo and Schwartz, 2014), who reported that walking on a treadmill for 4 min at a self-selected pace improved divergent (evaluated by the AUT) but not convergent thinking (evaluated by the compound remote-association test). It is unclear why walking affects divergent but not convergent thinking despite the observation that both divergent and convergent thinking are related to the functioning of the PFC (Kleibeuker et al., 2013; Yuan and Raz, 2014; Dajani and Uddin, 2015; Wu et al., 2015; Peña et al., 2019). One possibility might be that the impact of aerobic exercise on convergent thinking depends on post-exercise mood (Aga et al., 2021), which, however, is not affected by walking or stair-climbing at very light intensity. This possibility remains to be tested by future well-designed studies. Regarding the neurobiological basis of the divergent thinking-enhancing effect of walking and stair-climbing, another potential mechanism in addition to the PFC may be the release of dopamine in response to physical activity (Chen et al., 2016, 2017). This proposal is based on the finding that dopamine is relevant to cognitive flexibility (Klanker et al., 2013) and associative or reinforcement learning (Chen, 2015; Chen et al., 2015), two cognitive processes underlying divergent thinking (Benedek et al., 2012).

As an everyday life physical activity, climbing stairs at comfortable or fast paces and with or without carrying groceries or other loads, has been a recommended physical activity in many governmental guidelines (Japanese Ministry of Health Labour and Welfare [JMHLW], 2013; US Department of Health and Human Services, 2018). Few studies, however, have investigated the specific physical and mental health benefits of stair-climbing. Allison et al. (2017) reported that brief intense stair-climbing involving 3 × 20 s “all-out” efforts produced robust physiological changes, including increased heart rate and blood lactate. Stenling et al. (2019) reported that compared to after no exercise, subjects had increased heart rate and felt more energetic, less tense, and less tired after three 1-min stair-climbing sessions (with 1-min recovery in between). In this study, males but not females showed better switching performance and neither showed improved inhibitory control ability (Stenling et al., 2019). These results, however, need to be replicated because their sample size was small (*n* = 11 for males). Our current study adds novel evidence to the literature of stair-climbing that a brief very light intensity stair-climbing enhances divergent thinking. As it has been reported that regular walking is associated with improved divergent but not convergent thinking (Nakagawa et al., 2020; Chen et al., 2021), future research may further investigate if regular stair-climbing has similar cognitive benefits.

Our study also had several limitations. Firstly, we used stair-climbing in a natural, real-life setting as our intervention, the intensity of which therefore was heterogeneous for each subject. This is different from heart rate reserve or aerobic capacity reserve-based exercise prescriptions (American College of Sports Medicine [ACSM], 2013). It is possible that people with higher aerobic capacity tend to use stairs more often and for more flights, although they may be unable to choose which floor to use for work or living. Secondly, we only tested stair-climbing as a round-trip between the fourth and the first floors. The intervention was considered very light in intensity and it is possible that stair-climbing at this very light intensity was unable to effectively enhance divergent thinking in some subjects. Future research is thus required to test stair-climbing at higher intensities to see if it boosts divergent thinking in all subjects. Thirdly, we used but one common test for the evaluation of divergent and convergent thinking, respectively. To validate our results, future studies should also test the effect of stair-climbing on other popular tests of divergent and convergent thinking. Fourthly, we limited our subjects to those in their twenties in order to exclude the confounding effect of age and improve statistical power. This, however, also limits the generalization of our findings to other populations. Future studies with more diverse subjects are needed to test whether our findings generalize to other populations.

## Conclusion

In a randomized controlled trial with a within-subjects crossover posttest comparison design, we found that compared to using the elevator, a brief stair-climbing intervention involving a round-trip walking for three flights enhanced divergent thinking in a sample of healthy young adults. Subjects on average generated 61% more original uses after stair-climbing than after using the elevator. Furthermore, stair-climbing cost less time compared to using the elevator. Our findings suggest that stair-climbing may be an efficient and useful strategy for enhancing divergent thinking in everyday life.

## Data Availability Statement

The data that support the findings of this study are available from the corresponding author upon reasonable request.

## Ethics Statement

The studies involving human participants were reviewed and approved by Yamaguchi University Institutional Review Board. The patients/participants provided their written informed consent to participate in this study.

## Author Contributions

CC and SN: conceptualization. KM, CC, KH, NS, YO, HL, and AT: methodology. KM and CC: formal analysis and writing—original draft preparation. KM, CC, NS, and MH: investigation. YF and FH: resources. All authors contributed to writing—review and editing and have read and agreed to the published version of the manuscript.

## Conflict of Interest

The authors declare that the research was conducted in the absence of any commercial or financial relationships that could be construed as a potential conflict of interest.

## Publisher’s Note

All claims expressed in this article are solely those of the authors and do not necessarily represent those of their affiliated organizations, or those of the publisher, the editors and the reviewers. Any product that may be evaluated in this article, or claim that may be made by its manufacturer, is not guaranteed or endorsed by the publisher.

## Supplementary Material

Supplementary Figure 1 Schematic illustration of the round-trip path and the layout of the first and fourth floors used for the interventions.
