# Supplementary figures and images for: The Effect of Brief Stair-Climbing on Divergent and Convergent Thinking
Source: Front Behav Neurosci. 2022 Jan 28;15:834097. doi: 10.3389/fnbeh.2021.834097 (PMC8831728; doi:10.3389/fnbeh.2021.834097)

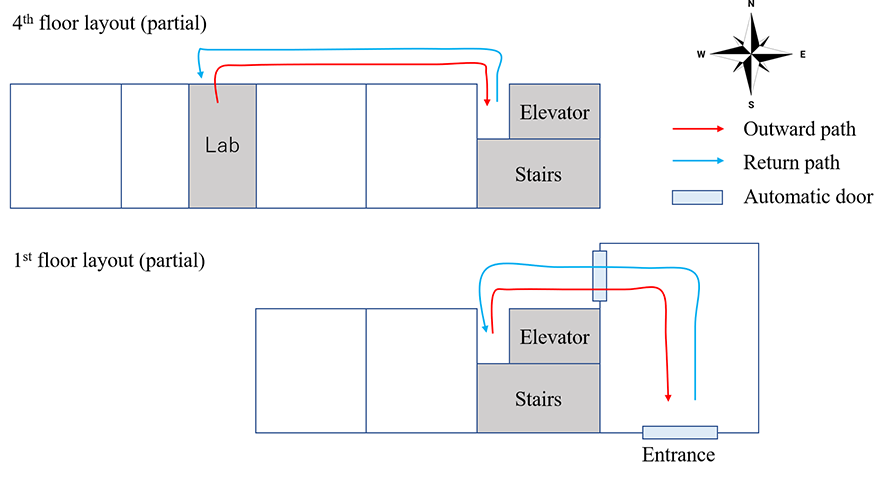

Supplement: Supplementary Figure 1 — Schematic illustration of the round-trip path and the layout of the first and fourth floors used for the interventions. [file Image_1.tif]
